# Perivascular macrophages in the CNS: From health to neurovascular diseases

**DOI:** 10.1111/cns.13954

**Published:** 2022-09-20

**Authors:** Li Zheng, Yunlu Guo, Xiaozhu Zhai, Yueman Zhang, Weijie Chen, Ziyu Zhu, Wei Xuan, Peiying Li

**Affiliations:** ^1^ Department of Anesthesiology, State Key Laboratory of Oncogenes and Related Genes Shanghai Cancer Institute, Renji Hospital, Shanghai Jiao Tong University School of Medicine Shanghai China

**Keywords:** Alzheimer's disease, neurological disease, perivascular macrophages, stroke

## Abstract

Brain perivascular macrophages (PVMs) are attracting increasing attention as this emerging cell population in the brain has multifaced roles in supporting the central nervous system structure, brain development, and maintaining physiological functions. They also widely participate in neurological diseases such as neurodegeneration and ischemic stroke. Moreover, PVMs have been reported to have both beneficial and detrimental effects under different pathological contexts. Advanced research technologies allowed the further in‐depth study of PVMs and revealed novel concepts in their origins, differentiation, and regulatory mechanisms. Deepened understanding of the roles of PVMs in different brain pathological conditions can reveal novel phenotypic changes and regulatory signaling, which might pave the way for the development of novel treatment strategies targeting PVMs.

## Graphical abstract

PVMs exert beneficial effect in Aβ clearance in Alzheimer's disease, but the production of reactive oxygen species (ROS) is harmful to the brain. In terms of hypertension, circulating AngII can stimulate ROS production through the activation of Atr1 on PVMs, resulting in cerebrovascular dysfunction. Moreover, PVMs mediate the deterioration of neurological impairment in the acute phase of stroke, despite the phagocytosis function in the early stage.

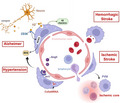

## 1 INTRODUCTION

Innate immune cells in the brain are increasingly recognized as an important player in maintaining brain homeostasis and the development of brain diseases.^1^, ^2^, ^3^, ^4^ In addition to the widely studied microglia in the brain parenchyma, non‐parenchymal border‐associated macrophages (BAMs) such as PVMs, are one type of innate immune cells in the brain that have also been shown to participate in brain development, maintenance of homeostasis, neurodegenerative diseases, ischemic stroke, and other processes.^5^ Understanding the functions of PVMs in cerebral steady‐state and disease progression can provide important insights for the development of treatment strategies for neurological diseases in the future. For this purpose, we summarize the latest research on PVMs in recent years, including the effects and modulatory mechanisms in neurodegeneration diseases, which are the novelties of this review.

## 2 DISCOVERY OF PVMs IN THE BRAIN

PVMs were first discovered in the 1980s by Mato et al^6^ using trypan blue and horseradish peroxidase injection into the ventricles, which were taken up by slender cells located in the perivascular space. They did a lot of research over the following several decades and found fluorescent granular perithelial (FGP) cells that could remove the metabolic waste of brain parenchyma with globular vacuolated inclusions in their cytoplasm,^7^ and incorporate lipids in circulation.^8^ Notably, FGP cells were distributed in the space around cerebral arterioles and venules, which was different from the pericytes embedded in the basement membrane of capillaries.^9^ At the same time, in 1988, Hickey et al also described the slender and glycoprotein ED2 positive “perivascular microglia” around the blood vessels.^10^ As microglia do not express ED2 (CD163),^11^ these perivascular cells were confirmed to be different from microglia. Thus, scientists then gradually recognized that PVMs are unique myeloid cells located in the brain perivascular Virchow‐Robin space (VRS).

It is now well‐accepted that BAMs are non‐parenchymal macrophages in the central nervous system (CNS) and located in the boundary regions including VRS, meninges, and choroid plexus.^12^ As the name suggests, PVMs are macrophages located in the perivascular VRS of the CNS. Specifically, the VRS refers to invaginations surrounding cerebral vessels, and distinct interfaces connecting blood, cerebrospinal fluid (CSF), and brain parenchyma.^13^, ^14^ PVMs exactly reside around arterioles and venules both in cortical and subcortical regions of the mouse brain.^15^ This special anatomical location of PVMs allows their direct contact with blood vessels and parenchyma, providing structural and functional support for the blood–brain barrier (BBB).^16^ PVMs are also essential in maintaining brain homeostasis. In recent years, increasing evidence support that PVMs are key components of the brain resident immune system and are involved in number of pathological processes, especially in neurodegeneration and ischemic stroke.^7^, ^8^, ^17^, ^18^, ^19^, ^20^, ^21^, ^22^, ^23^, ^24^

## 3 DEVELOPMENT AND DIFFERENTIATION OF PVMs

### 3.1 New views on the origin of PVMs

The origin of PVMs has been discussed for a long time.^25^, ^26^, ^27^ For decades, it was thought that all PVMs came from circulating monocytes and were updated frequently.^10^, ^28^, ^29^, ^30^ However, this conclusion was questioned later, because it was based on the experiments of full‐body irradiation and bone marrow transplantation in rodents. Both of the experiments may cause the overexpression of chemokines and the destruction of the BBB, resulting in the entry of the bone marrow‐derived monocytes into the CNS.

New views on the origin and renewal of PVMs have been put forward through large‐scale single‐cell RNA‐sequencing (scRNA‐seq), parabiosis, fate‐mapping, and in vivo imaging. In general, PVMs and microglia are both of prenatal origin and PVMs have a closer transcriptional relationship with microglia than monocytes. It is now well accepted that PVMs arise from early erythro‐myeloid progenitors in the yolk sac, which migrate into the brain in the early stage of the embryo.^31^ Using scRNAseq, two phenotypically and transcriptionally distinct macrophages, which separately differentiate into microglia and PVMs, can be found and distinguished in the developing brain and the yolk sac, indicating an early separation into two different populations. In addition, the development of PVMs is independent of TGF‐β, while microglia need TGF‐β for development.^32^ In normal conditions, PVMs are a stable cell population with a long lifespan and self‐renewal ability after birth. When the PVMs are depleted by laboratory methods, they can be replenished from bone marrow‐derived monocytes.

### 3.2 Regulatory mechanisms of the differentiation of PVMs

The development and normal functions of PVMs are regulated by many factors, including transcription factors and cytokines. We summarize several key influencing factors modulating PVMs.

#### 3.2.1 Transcriptional regulation

In recent years, transcription factors have been shown as the key determinants in the orchestration of myeloid identity and differentiation fates.

PU.1 is a member of the large family of E‐twenty six transcription factors and it is the product of the oncogene Spi1. PU.1 exists in almost all myeloid‐specific and many lymphoid‐specific gene regulatory sequences, and most PVM‐specific enhancers contain binding domains for PU.1.^33^ The absence of Spi1 in mice can lead to fatal defects in fetal liver and/or newborn hematopoiesis, including the complete loss of macrophages.^34^, ^35^ The more recent analysis has demonstrated that the absence of PU.1 impairs the repopulation capacity of the hematopoietic stem cells (HSCs), impeding their differentiation into the common myeloid progenitors and the common lymphoid progenitors.^36^, ^37^, ^38^

The survival of brain PVMs also depends on transcription factor c‐MAF, which is part of the large Maf family of transcription factors. Conditional knockout of c‐MAF in macrophage lineages will cause ablation of PVMs in the CNS.^39^

MAFB also belongs to the Maf family, with the function of controlling the proliferation rate of PVMs through the epigenetic regulation of self‐renewing genes.^40^, ^41^ Beyond that, MAFB is also able to limit the ability of macrophage colony‐stimulating factor (M‐CSF) in differentiating HSCs to PVMs.^39^, ^42^

Interferon regulatory factor 8 (IRF8) is critically involved in driving the maturation and diversity of brain macrophages. The deficiency of IRF8 will cause alternations in PVMs development and function.^43^, ^44^

#### 3.2.2 The cytokines that regulate myeloid cell fate

M‐CSF, also known as CSF‐1 is the major cytokine modulating macrophages' proliferation, differentiation, and functional regulation.^45^ M‐CSF is produced by a variety of stromal and epithelial cells. It transmits signals through M‐CSF receptors (M‐CSFR/CSF‐1R/CD115). It has been shown that M‐CSF can guide the myeloid fate of HSCs by inducing PU.1,^46^ and it is important in establishing and maintaining tissue‐resident macrophage pools.^47^ In addition to influencing the differentiation and maintenance of macrophages, M‐CSF can also stimulate the survival and self‐renewal of macrophages in steady‐state and inflammation. Moreover, it is involved in the polarization of macrophage activation.^48^, ^49^

Interferon‐gamma (IFN‐γ) can interact with PVMs by upregulating major histocompatibility complex (MHCII) and B7 coreceptor expression, and shift PVMs from anti‐inflammatory to proinflammatory cytokine profiles.^50^

The above evidence suggests that IFN‐γ has a direct effect on the phenotypic switch of PVMs, while M‐CSF could also be an important determinant cytokine of PVM cell fate and phenotypic polarization. However, further studies are warranted to identify the roles of different cytokines in the regulation of PVMs.

## 4 CHARACTERIZATION AND RECOGNITION OF PVMS IN THE BRAIN

PVMs are characterized by their anatomical localization, phagocytosis ability, and molecular markers. Firstly, PVMs can be recognized by their specific locations. Nowadays, people believe that PVMs are located in the VRS between the vascular basement membrane on the abluminal side and the glial limitans of the brain parenchyma.^15^ And they belong to a group of distinct myeloid cells.

Secondly, the phagocytosis function of PVMs can be utilized for their identification. For example, by intravenous injection of fluorescence‐labeled dextran, PVMs can be clearly visualized due to their phagocytosis of the fluorescent dextran.^16^, ^51^ Interestingly, their phagocytosis ability can also be utilized to achieve PVMs depletion by intraventricular injection of clodronate (CLO)‐containing liposomes.^16^, ^17^, ^52^, ^53^

**FIGURE 1:**
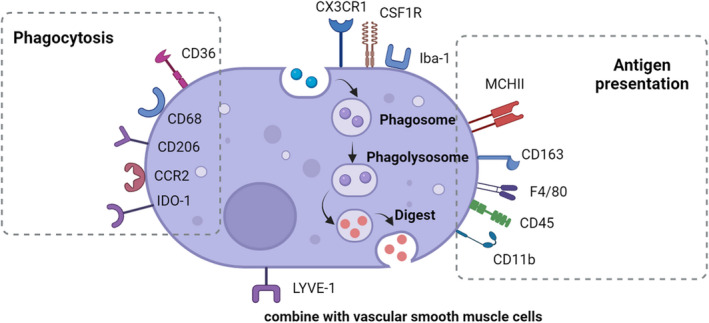
Characteristic markers of PVMs in the brain. All leukocytes express CD45 and CD11b, and besides, the brain‐resident myeloid cells express Cx3cr1, CSF1R, and Iba‐1. Conventional PVMs markers include CD163, Lyve‐1, and CD206. Compared to microglia, PVMs have higher expression levels of CD45, F4/80, Iba‐1, and MHCII. A variety of markers have been confirmed to be related to the phagocytosis of PVMs such as CD36, CD68, CD206, CCR2, and IDO‐1. And some markers have been confirmed to participate in the antigen presentation such as CD163, CD45, CD11b, MCHII, and F4/80. LYVE‐1 is a receptor of hyaluronan, controlling the expression of collagen in vascular smooth muscle cells.

Importantly, PVMs express a number of markers that can be distinguished from microglia (Table 1, Figure 1). Besides CD45 and CD11b, the brain‐resident myeloid cells all express fractalkine receptor (Cx3cr1), CSF1R, and allograft inflammatory factor 1 (Iba‐1).^5^ PVMs have higher expression levels of CD45, F4/80, Iba‐1, and MHCII,^5^, ^54^ and lower levels of Cx3cr1 compared to microglia.^55^ PVMs are negative for microglial‐specific markers such as transmembrane protein 119 (TMEM119), sialic acid‐binding immunoglobulin‐like lectin H (Siglec‐H), P2Y purinoceptor 12 (P2RY12), Sal‐like protein 1 (Sall1), Sal‐like protein3 (Sall3), or ANXA3.^5^, ^56^, ^57^, ^58^, ^59^ Instead, brain PVMs express nonconventional macrophage markers such as Siglec1 (CD169), which is absent in microglia.^60^ These features can be used to distinguish microglia and PVMs.

**TABLE 1:** Differentiation markers of PVM and microglia

Markers	Function	PVM	Microglia	References
CD45	T cell and B cell receptor‐mediated activation	++	+	[5]
CD11b	Cell adhesion, apoptosis, chemotaxis	+	+	[66, 67]
Cx3cr1	Fractalkine receptor	+	++	[55, 68]
CSF1R	Csf1 receptor	+	+	[69, 70]
Iba‐1	Still unknown	++	+	[51, 71]
F4/80	Interact with circulating immune cells	++	+	[34, 72]
MHCII	Antigen presentation	++	+	[35, 54]
CD68	Lysosomal protein	+	+/−	[73]
CD163	Endocytosis; scavenger receptor; antigen presentation	+	−	[61, 62, 74]
Lyve‐1	Hyaluronan receptor, controlling the expression of collagen in vascular smooth muscle cells	+	−	[75]
CD206	Mannose receptor; endocytosis	+	−	[5, 10, 40]
TMEM119	Still unknown	−	+	[41, 59, 60, 76]
P2RY12	Nucleotide receptor	−	+	[41, 77]
SALL1	A zinc‐finger transcription factor	−	+	[58]
CD169	Sialoadhesin, endocytosis	+	−	[78]
ANXA3	Belongs to the structurally‐related annexin protein family	−	+	[56]
Siglec‐H	Still unknown	−	+	[57]

Some conventional PVMs markers could differentiate PVMs with microglia, such as CD163, lymphatic vessel endothelial hyaluronan receptor‐1 (Lyve‐1), and CD206. CD163 is expressed in PVM and monocytes, but not in microglia.^61^, ^62^ The mannose receptor CD206 is only expressed in BAMs, which can be used to distinguish PVMs from microglia and monocytes. However, transient high expression of CD206 can be found in a subpopulation of microglia and infiltrating macrophages after a brain injury such as stroke and brain trauma.^63^, ^64^ The monoclonal antibody 5D3 can be used to localize the expression of mannose receptors on PVMs in normal CNS and various models of brain pathology with good specificity.^65^ Based on these different markers, a binary transgenic model has recently been used to dissect microglia and PVMs for further separate study.^55^

In summary, so far there is no single marker of PVMs with good specificity and sensitivity. The expressions of PVM markers combined with their anatomical location and phagocytic feature may be an ideal and reliable way for identification.

## 5 PVM, THE FIRST LINE “FIREWALL” MAINTAINING THE HOMEOSTASIS OF THE CNS

Under physiological conditions, there are different types of macrophages in the CNS, performing their functions and maintaining the homeostasis of the brain. As an important part of BAMs, PVMs act as the first “firewall” in the CNS because of their special anatomical location and innate immune functions. Paragraphs outlined below discussed PVM functions in the regulation of BBB permeability, immune regulation, phagocytosis, and lymphatic clearance.

### 5.1 PVMs regulate the integrity of BBB

The integrity of BBB is essential for the brain to maintain its homeostasis and is an important anatomical structure that mediates the entry of the essential components into the brain parenchyma and also prevents the invasion of pathogens and blood‐derived harmful substances.^79^, ^80^, ^81^, ^82^ It is well known that brain capillary endothelial cells and their tight junctions play key roles in maintaining the BBB permeability.^83^, ^84^, ^85^ Meanwhile, the participation of PVMs has been recognized recently.^86^, ^87^, ^88^ Under physiological conditions, the microvasculature of the area postrema (AP) has a less restrictive BBB than is found in other CNS areas due to the lack of tight junction.^20^ In this case, PVMs in this area can isolate 10–70 kDa serum proteins from the blood and combine the laminin layer, further helping to restrict the entry of solutes above 10 kDa into the parenchyma.^86^ In addition, using the cell culture model of the BBB, Zenker et al^89^ found that the transendothelial electrical resistance of post‐confluent brain capillary endothelial cells was significantly increased by coculture with blood‐derived macrophages, which could partly indicate that PVMs can be involved in the maintenance of BBB permeability.

The effect of PVMs on BBB seems to be two‐edged. In normal condition, they are necessary for the maintenance of BBB, but in the case of CNS injury and neuroinflammation, they seem to mediate the damage of BBB. Most recently, PVMs have been confirmed to participate in BBB disruption through the release of cytotoxic mediators under malaria.^90^ What's more, another review analyzed literature from 2000 to 2021 and revealed that PVMs could cause BBB damage in Alzheimer's disease (AD).^91^

Taken together, the emerging literature suggests that PVMs modulate the integrity of BBB. Future research should investigate the specific regulatory mechanisms in this process.

### 5.2 Immune regulation and antigen presentation of PVMs

In a steady‐state, there are few circulating immune cells in the brain, but a variety of immune cell subtypes can infiltrate into CNS in the case of inflammation, trauma, autoimmune diseases, and so on. Classical immunostimulation with lipoteichoic acid from gram‐positive bacteria and lipopolysaccharide (LPS) from gram‐negative bacteria can lead to the proliferation of PVMs. PVMs show phenotypic plasticity in many homeostatic and pathological situations. PVMs have been confirmed that they can exert proinflammatory polarization (M1) in neurological diseases including ischemic injury in mice.^15^ We have reason to believe that PVMs may also have different types of classification as a kind of macrophages. However, PVMs activation status may also not be simply classified into M1/M2 due to their complex nature. Thus, further research is warranted to identify different phenotypes of PVMs.

Previous studies have confirmed that PVMs can act as antigen‐presenting cells (APCs) under certain pathological circumstances,^21^, ^22^, ^23^, ^24^ they are essential in the CNS as APCs both in vitro and in vivo.^8^, ^33^, ^92^ One of the important characteristics of APCs is the expression of MHCII.^93^ PVMs express MHCII and can present antigen to lymphocytes in an experimental autoimmune encephalomyelitis (EAE) model.^10^ MHCII^high^ PVMs can be observed under pathological conditions, such as transient middle cerebral artery occlusion (tMCAo), EAE in rodents, and multiple sclerosis (MS) from the human autopsy.^10^, ^62^, ^94^ A recent study also showed it was PVMs that presented antigens to CD8^+^ T cells in experimental cerebral malaria.^90^ Thus, PVMs also have the function of bridging innate and adaptive immunity in the CNS.

### 5.3 The phagocytic ability of PVMs

Under physiological conditions, PVMs are considered to be the scavenger cells and surveillant cells of the brain, as they occupy the ideal position of monitoring and removing potentially harmful substances. As mentioned before, PVMs were first discovered due to their uptake of dyes.^6^ Researchers also discovered that this kind of cells can remove metabolic waste from brain parenchyma, and bind to lipids in the circulation to clear the lipid deposition increased in aging animals.^7^, ^8^ PVMs have also been shown to gobble up metabolic waste and cellular breakdown products in some brain diseases such as experimental subarachnoid hemorrhage and cerebral amyloid angiopathy.^17^, ^95^

The phagocytosis property of PVMs has been proved relevant to the markers expressed on PVMs such as CD68, CD163, CD206, and IDO‐1. CD68 is a lysosomal protein that promotes intracellular lysosomal function.^73^ CD163 is a scavenger receptor (SR) protein that recognizes and endocytoses the hemoglobin/haptoglobin complexes and participates in antigen presentation.^74^ CD206 is a mannose receptor that may be involved in the scavenging effect.^65^, ^96^ IDO‐1 is an immunosuppressive enzyme that increases cellular phagocytic capacity and might suppress the overactivation of inflammatory response.^97^

In summary, the phagocytic ability of PVMs under physiological and also pathological conditions is of vital importance for maintaining brain homeostasis by clearing exogenous substances, endogenous metabolic waste, and cellular debris.

### 5.4 Lymphatic clearance of PVMs

The brain lymphatic system has momentous physiological functions: excreting interstitial fluid (ISF) to the nearby lymph nodes from the brain parenchyma, maintaining water and electrolyte balance of the ISF, clearing metabolic waste, and reabsorbing macromolecular solutes^98^, ^99^; and communicating with the immune system, modulating immune surveillance, and the inflammatory response. The cerebral lymphatic drainage system is composed of a basement membrane‐based perivascular pathway,^100^ a brain‐wide paravascular glymphatic pathway,^19^ and some CSF drainage routes including sinus‐associated meningeal lymphatic vessels^18^, ^101^, ^102^, ^103^ and olfactory/cervical lymphatic routes.^104^, ^105^ Given their close relationship to vessels, PVMs may facilitate the first two pathways.

The “intramural perivascular drainage pathway” (IPAD) is a pathway in the vessel wall of the tunica media which is composed of vascular smooth muscle cells (VSMCs).^100^, ^106^ Injection tracers into the caudate putamen were found to enter the arterial wall and travel along the intercellular spaces among the VSMCs. PVMs can promote the clearance of the IPAD by taking up 2 nm to 1 μm particles. Furthermore, PVMs mediate the speed of IPAD by regulating the contraction and relaxation of VSMCs, and it is found that the increase in age will lead to a significant slowdown of IPAD.^107^ The glymphatic pathway also involves “paravascular space.”^19^ CSF enters the parenchyma along paravascular spaces which surround penetrating arteries and the brain ISF is cleared via paravenous drainage pathways.

PVMs can facilitate lymphatic drainage in the CNS in the above two ways mentioned above. The exact role of PVMs in these pathways is worth further study and discussion.

In summary, because of the special anatomical location in the brain, PVMs can directly contact blood, CSF, and brain parenchyma. PVMs exert phagocytic function and clear metabolic waste. PVMs can also act as APCs to recruit circulating immune cells into CNS. In addition, they provide structural and functional support for BBB and lymphatic clearance, which is important for the maintenance of brain homeostasis and normal functions.

## 6 PVMs IN NEUROLOGICAL DISEASES

As mentioned above, PVMs are vitally important in maintaining brain homeostasis. In the past few years, more and more evidence supported the theory that PVMs are widely involved in neurological diseases (Table 2). Here, we mainly focused on AD, hypertension‐associated neurovascular dysfunction, and stroke.

**TABLE 2:** PVMs in various neurological diseases

Disease	Model	PVMs manipulation	Effect of PVMs	References
AD	Tg2576 mice (Aβ regional perfusion on brain cortex, Aβ i.v. administration, and Aβ overexpression)	CLO/bone marrow chimeras (CD36^−/−^ and Nox2^−/−^)	PVMs are involved in Aβ‐induced neurovascular dysfunction through oxidative stress	^16^
	TgCRND8 mouse model of AD	CLO/chitin	PVMs promote Aβ clearance	^17^
	J20 transgenic mice	SR‐B1^+/−^ and SR‐B1^−/−^ mice	PVMs express SR‐B1, promote Aβ clearance	^53^
Ischemic stroke	Alcohol‐exposed Swiss mice	CLO	PVMs Participate in the initiation of neurovascular inflammation and the aggravation of inflammatory responses after a secondary insult	^108^
	Patients with focal cerebral ischemia	N/A	PVMs proliferate and express COX‐1 mediate tissue remodeling	^109^
	Wistar rats	Bone marrow chimeras	PVMs proliferate and migrate to the brain parenchyma	^78^
	Sprague‐Dawley rats with MCAO	CLO	PVMs recruit granulocyte, increase BBB permeability, promote neurological dysfunction	^110^
SAH	CD1 mice	CLO	PVMs uptake erythrocyte and contribute to neuroinflammation	^95^
Hypertension	BPH/2J	CLO/bone marrow chimeras (At1r^−/−^ and Nox2^−/−^)	PVMs mediate neurovascular dysfunction through oxidative stress	^51^
	SHRSP/Izm rats	N/A	PVMs participate in collagen deposition, contribute to the atherosclerotic changes under hypertension	^111^
	SHRSP	CLO	PVMs contribute to the development of hypertension via sympathetic activation	^112^

### 6.1 PVMs and Alzheimer's disease

AD is the main cause of cognitive impairment in the elderly, pathologically characterized by extracellular deposition of the amyloid‐β (Aβ) and intracellular aggregates of the microtubule‐associated protein tau (neurofibrillary tangles).

The brain is highly dependent on the continuous regulation of cerebral blood flow (CBF) to transport oxygen and glucose for the brain's energy needs. Not surprisingly, alternations in cerebral perfusion can cause brain dysfunction and cognitive impairment. A large number of studies have shown that Aβ disrupted cerebral microcirculation. Aβ inhibits the increase of CBF correlated to synaptic activity and interferes with endothelial function.^113^, ^114^, ^115^, ^116^, ^117^, ^118^, ^119^, ^120^ The brain Aβ is released to extracellular space during synaptic activity^121^ and reaches the VRS.^122^ In this space, PVMs are in direct contact with Aβ, which mediates special pathophysiological processes.

Previous studies have suggested that besides microglia, the phagocytosis of PVMs is essential for Aβ clearance. Depletion of PVMs is related to the vascular accumulation of Aβ 42 and the severity of cerebral amyloid angiopathy.^17^ Scavenger receptors (SRs) are widely expressed by microglia/macrophages and are able to bind a diverse array of endogenous and foreign molecules, thus playing critical roles in the phagocytosis of these cells. The phagocytic function of PVMs was found to be regulated by the high‐density lipoprotein receptor (SR class B type 1, SR‐B1) on PVMs that regulates the flow of cholesterol. The exhaustion of SR‐B1 can impair PVMs response to Aβ and accelerate the formation of cerebrovascular and also parenchymal amyloid plaques in the cerebral cortex and hippocampus of mice, thus aggravating cognitive impairment.^53^

**FIGURE 2:**
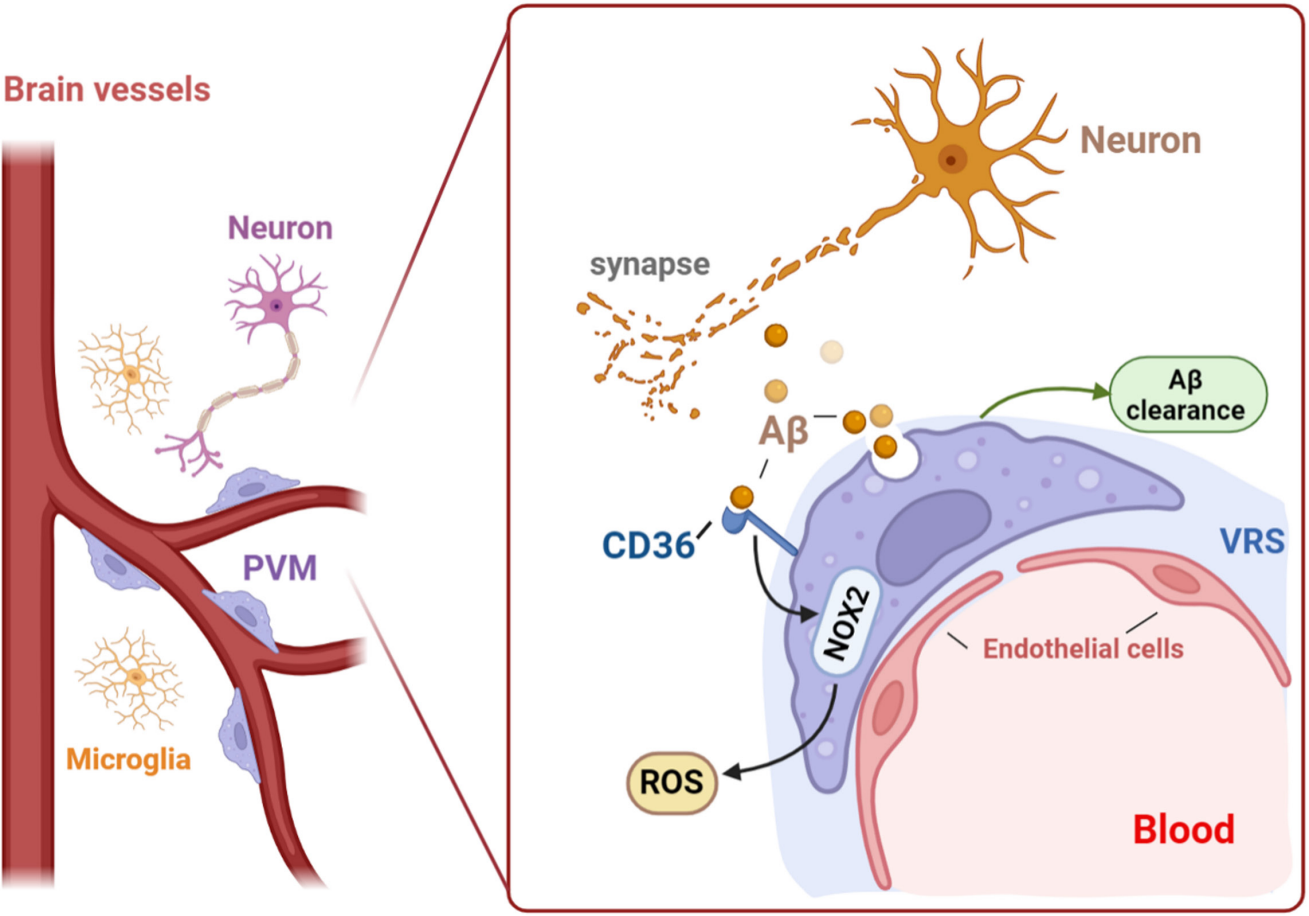
The role of PVMs in AD PVMs has both positive and negative effects on the development of AD. AD is characterized pathologically by extracellular deposition of the amyloid‐β peptide (Aβ) in amyloid plaques and intracellular aggregates of the microbubble‐associated protein tau. A‐β is released to extracellular in the process of synaptic activity and reaches the VRS, where the phagocytosis function of PVMs is essential for A‐β clearance. Besides, PVMs are involved in A‐β‐induced neurovascular dysfunction through CD36‐mediated oxidative stress. CD36 binds A‐β and leads to NADPH oxidase 2 (Nox2)‐dependent production of reactive oxygen species (ROS).

Nevertheless, in addition to the above beneficial effects, PVMs also take part in the negative side of AD development. PVMs are involved in Aβ‐induced neurovascular dysfunction through CD36‐mediated oxidative stress. CD36 binds Aβ and leads to NADPH oxidase 2 (Nox2)‐dependent production of reactive oxygen species (ROS).^122^, ^123^ Selective depletion of PVMs can abrogate the neurovascular dysfunction and vascular oxidative stress induced by Aβ^16^ (Figure 2).

In addition, aging reduces the activity of PVMs and causes cell dysfunction, along with the alteration of the structure and distribution of PVMs. Mato et al found the amount of lipid precipitation in the cytoplasm increased significantly with age.^7^ In PVMs of young subjects, most inclusion bodies are round, uniform in content, and high in electron density. However, in elderly subjects, PVMs show many enlarged inclusion bodies and often display a honeycomb structure.^7^ At the same time, in both elderly experimental animals and humans, the swollen PVMs often appear at bifurcations of cerebral arterioles and compress arterioles, which contribute to the disturbance of cerebral blood flow.^7^

To sum up, the bi‐directional regulation of Aβ by PVMs reminds us not to simply block or boost PVMs in AD. Instead, it is meaningful for the future study on the regulation of PVMs to take advantage and avoid the reverse effects of AD.

### 6.2 PVMs and hypertension‐associated neurovascular dysfunction

The health of the cerebrovascular system is of vital importance to the functional and structural integrity of the brain.^51^ Remarkably, hypertension can disrupt the cerebrovascular system, which is the basis of neurovascular cognitive impairment.^124^, ^125^, ^126^, ^127^ Recent studies suggested that PVMs take part in the modulation of neurovascular and cognitive dysfunction associated with hypertension.

PVMs mediate cerebral neurovascular dysfunction in hypertension through the angiotensin type 1 receptor (Atr1).^51^ In hypertension, the elevated Ang II can reach the perivascular space through the damaged BBB, then activate Atr1 on PVMs, resulting in NOX2‐dependent ROS production, finally leading to cerebral vascular dysfunction and cognitive dysfunction.^51^ Another study demonstrated that by depleting most of the PVMs and all the microglia in Ang II‐induced hypertensive mouse model, short‐term memory impairment can be prevented.^128^ What's more, PVMs contribute to the development of hypertension, both the number and activity of the PVMs are increased by the stimulation of proinflammatory cytokine.^112^, ^129^ Then, prostaglandin E2 (PGE2) produced by PVMs enters the brain parenchyma, resulting in sympathetic activation and blood pressure elevation.^112^ Interestingly, in vitro study, it is confirmed that extracellular PGE can promote microglia to produce more PGE and COX‐2.^130^ All of the above processes will play momentous roles in the development of CNS diseases. Based on these studies, we speculate whether PVMs and microglia have a synergistic effect in Ang II‐mediated hypertensive cerebrovascular disease worth deeper study.

**FIGURE 3:**
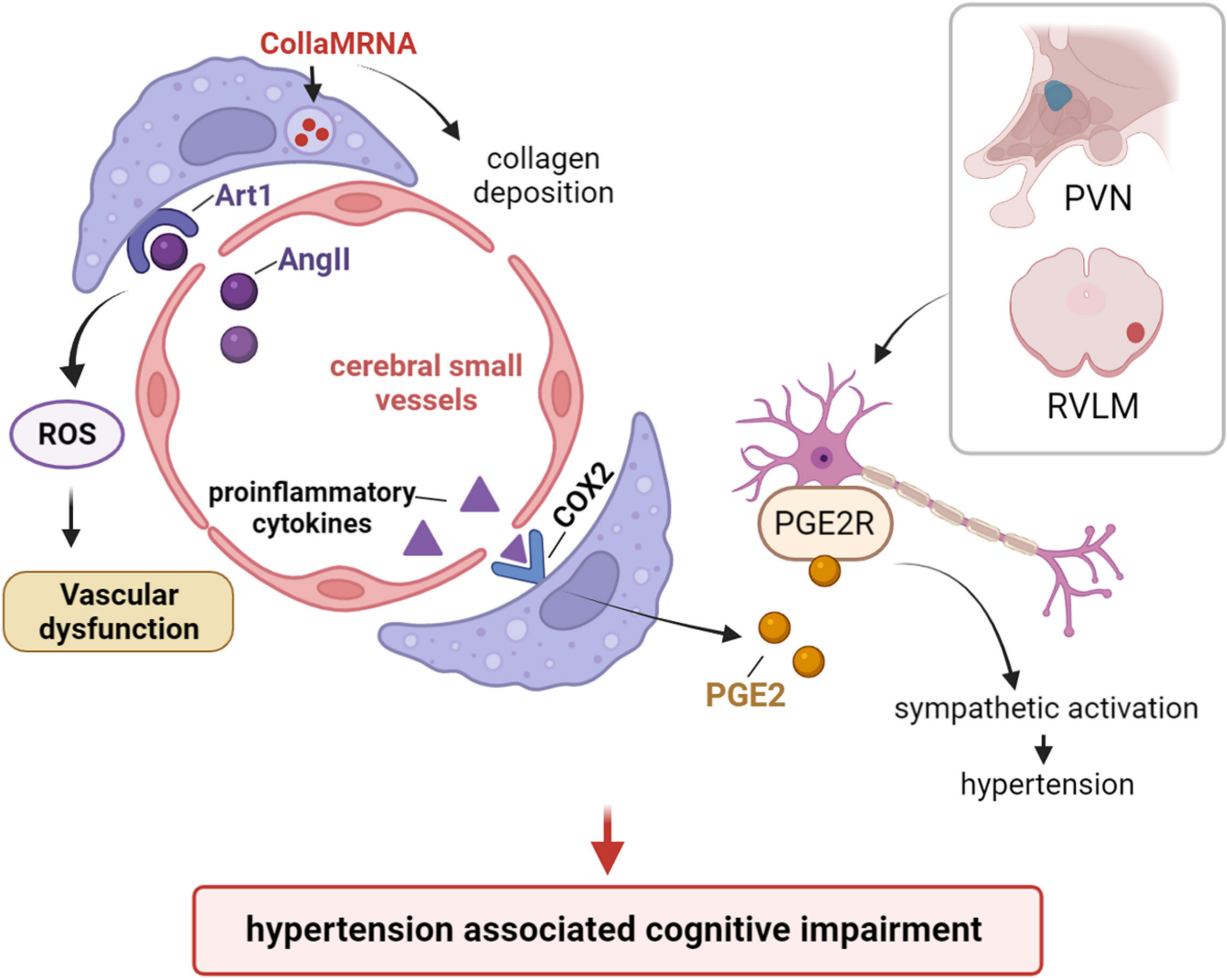
The role of PVMs in hypertension‐associated cognitive impairment. PVMs can mediate hypertension‐associated neurovascular and cognitive dysfunction in many ways. Expressing Col1a1MRNA, PVMs mediate the production of type I collagen. Collagen deposits around the cerebral small vessels induces the change of atherosclerosis during hypertension. Circulating AngII can activate Atr1 on PVMs, resulting in NOX2‐dependent ROS production, which then leads to cerebral vascular dysfunction and cognitive impairment. PVMs produce PGE2 through COX‐2, PGE 2 enters the brain parenchyma and activates PGE2 receptors on the PVN and the rostral ventrolateral medulla (RVLM), leading to sympathetic activation and an increase in blood pressure.

PVMs are involved in the process of cerebrovascular remodeling in hypertension as well. The remodeling and progression of atherosclerosis in hypertension contain fibrosis and the production of type I collagen around the cerebral arterioles.^111^ PVMs around the cerebral small vessels express Col1a1 mRNA, which mediates the production of type I collagen, makes collagen deposition around the cerebral small vessels, and participates in the change of atherosclerosis during hypertension^111^ (Figure 3).

It can be seen that PVMs participate in neurovascular and cognitive dysfunction related to hypertension from many aspects. Hence, a deeper understanding of how PVMs influence the remodeling of the cerebrovascular system may help to optimize the therapies for the recovery and rehabilitation of related diseases.

### 6.3 Stroke and PVMs

Stroke remains the second leading cause of death and the third leading cause of death and disability worldwide in 2019.^131^ Therefore, it is important and necessary to clarify the mechanisms of the stroke to lighten the burden on families and even the whole world. It is increasingly recognized that PVMs play an important role in the acute inflammatory phase and secondary injury after stroke.

According to statistics, ischemic stroke constituted 62.4% of all strokes.^131^ Researchers have confirmed the functions of PVMs in ischemic stroke in many ways. For instance, PVMs participate in the neuropathological process through cell proliferation and migration to the ischemic brain parenchyma, as PVMs are found highly accumulated in peri‐infarction areas and in the developing necrotic core area in the early stage after cerebral infarction, and the number continues to increase until several months after stroke.^109^ More than that, PVMs also up‐regulate the expression of COX‐1, which plays an important role in the pathophysiology of acute ischemic inflammation, tissue remodeling, and secondary injury after stroke. Aside from acting as direct proinflammatory cells, PVMs can also participate in granulocyte recruitment by upregulating the expression of leukocyte chemo‐attractants.^110^ Moreover, PVMs have the function to elevate the expression of VEGF, increasing the permeability of pial and cortical blood vessels, and deteriorating neurological impairment in the acute phase of stroke.^110^ These results were also found in postmortem brain samples from ischemic stroke patients.^78^

A current study revealed that heavy drinking of alcohol (> = 6 standard drinks/day) is an independent risk factor associated with worse outcomes in ischemic stroke patients.^108^ PVMs are activated in mice with chronic alcohol exposure, and the inflammation significantly increased after a secondary insult (ischemic stroke or LPS challenge). Depletion of PVMs can block the alcohol‐induced aggravation of ischemic lesions.

**FIGURE 4:**
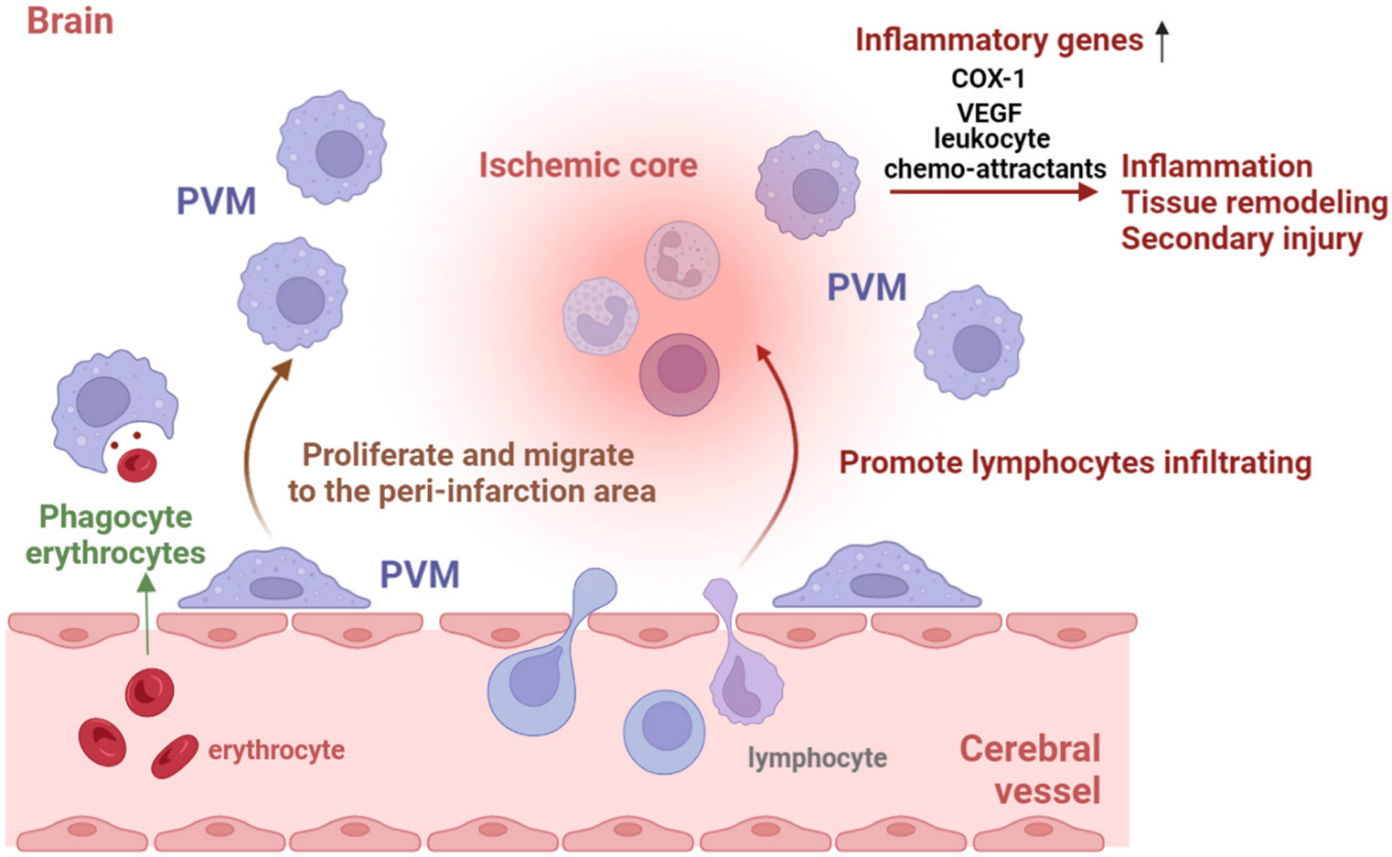
The main modulatory mechanisms of PVMs after stroke onset. After the stroke, PVMs proliferate and migrate to the lesion core, up‐regulating the expression of many inflammatory genes including COX‐1, VEGF, leukocyte chemo‐attractants, and promoting lymphocytes enter into the CNS. Although PVMs may still show harmful effects in the long run, they are the main cells responsible for phagocyting erythrocytes and their decomposition products in the early stage.

Subarachnoid hemorrhage (SAH) is a subtype of stroke and constituted 9.7% (about 1.18 million) of all strokes.^131^ As erythrocytes are damaged, there are many decomposition products such as bilirubin, heme, and free iron released into the CSF, which can cause inflammation, vasoconstriction, and direct cellular injury.^132^, ^133^, ^134^, ^135^ After SAH, erythrocytes enter the perivascular space, where they can interact with PVMs. Recent studies have found that erythrocytes are mainly removed by PVMs rather than microglia; however, the depletion of PVMs with CLO can decrease inflammation around arterioles and improve prognosis after SAH (Figure 4). This contradictory result is probably due to the reduced inflammatory burden after PVM depletion counteracting the negative effect of increased breakdown waste. It can be seen that although PVMs play a role in the phagocytosis of damaged erythrocytes and their decomposition products, PVMs may still show harmful effects in the long run.

To sum up, as a part of the brain innate immune cell population, PVMs are the important guardians of CNS homeostasis. Given the functional similarity of PVMs and microglia, whether PVMs display sex difference as in microglia is not clear.^136^, ^137^ The current literature is lacking on this issue. A deep understanding of how PVMs participate in the pathological mechanism of CNS diseases will be helpful to the development of treatment strategies.

## 7 CURRENT KNOWLEDGE GAPS AND FUTURE PERSPECTIVES

Growing evidence is suggesting the critical role of PVMs in maintaining brain hemostasis and regulating the progression of various neurological diseases. However, there are still a lot of unknowns and obstacles in the PVM research field. Here, we outline some of the key issues that need to be resolved.

1. The key regulatory genes and the underlying regulatory mechanisms of PVMs' differentiation, phenotypic switch, and cell fate under different disease contexts remain largely unknown.
2. The research findings from animal models are not able to fully reflect the changes of PVMs in the human body. The imaging techniques of PVMs in human is still underdeveloped.
3. Currently, there is no specific PVM targeted strategy that could allow precise manipulation of PVMs. Delivery techniques targeting PVMs are highly warranted for future PVM‐associated treatments.

Collectively, as important brain innate immune cells, the role of PVMs in the brain is emerging. Further research is warranted to expand the knowledge of the regulatory mechanisms of PVMs and the role of PVMs in various brain pathologies.

## CONFLICT OF INTEREST

The author(s) declared no potential conflicts of interest with respect to the authorship, and/or publication of this article. Peiying Li is an Editorial Board member of CNS Neuroscience and Therapeutics and a co‐author of this article. To minimize bias, they were excluded from all editorial decision‐making related to the acceptance of this article for publication.

## Data Availability

Data sharing is not applicable to this article as no new data were created or analyzed in this study.
